# Functional anatomy of the lacrimal gland in African black ostrich *Struthio camelus domesticus* in the embryonic and postnatal period

**DOI:** 10.4102/ojvr.v82i1.872

**Published:** 2015-03-25

**Authors:** Joanna Klećkowska-Nawrot, Karolina Goździewska-Harłajczuk, Renata Nowaczyk, Krzysztof Krasucki

**Affiliations:** 1 Department of Animal Physiology and Biostructure, University of Environmental and Life Sciences, Poland; 2 Department of Descriptive and Clinical Anatomy, Centre of Biostructure Research, Medical University of Warsaw, Poland

## Abstract

The aim of the present study was morphological and histochemical analysis of the lacrimal gland (LG) in African black ostrich *Struthio camelus domesticus* in the embryonic and postnatal period. Studies were conducted on 50 ostriches aged between the 28th day of incubation until 7 months old. Tissue sections were stained with haematoxylin and eosin, Azan trichrome, periodic acid-Schiff, Alcian blue pH 2.5, aldehyde fuchsin and Hale's dialysed iron. The LG in ostrich was classified as a tubulo-acinar type. The primordia of the lobes were determined in the LG structure on the 28th day of incubation, whilst the weakly visible lobes with acini and tubules were observed on the 40th day of incubation. Morphometric studies of the LG showed steady growth, characterised by an increase in both length and width. Histometric measurements of lobe size showed little difference between the first, second and third age groups, whilst in the fourth age group a marked increase in size of lobes was observed. The study showed that, apart from morphological changes, during the growth of the LG the character of acid mucopolysaccharides changed. Sulphated acid mucopolysaccharides were indicated, particularly with aldehyde fuchsin (AF) staining in the fourth age group. The Hale's dialysed iron (HDI) staining showed a low concentration of carboxylated acid mucopolysaccharides in the first and second age groups and a higher concentration in the third and fourth age groups. Periodic acid-Schiff staining (PAS)-positive cells were observed in each age group, but only a small number of cells with a weakly PAS-positive reaction were demonstrated in the first age group.

## Introduction

The lacrimal and Harderian glands are the main orbital glands belonging to the lacrimal apparatus (International Committee on Veterinary Gross Anatomical Nomenclature 2012; Jordan 1990; Paynter 1993). Both glands have been described in amphibians, reptiles, birds and mammals (Sakai 1988; Shirama & Hokano 1992). The anatomical structure of these glands is characterised by great variability (Chieffi Baccari, Di Matteo & Minucci 1992; Dimitrov 2011a, 2011b; Rehorek *et al*. 2005; Schechter, Warren & Mircheff 2010). In birds the lacrimal gland (LG) is less developed and of smaller size than the Harderian gland (Dimitrov 2011a; Dimitrov & Genchev 2011). The LG is located within the peri-orbita in the dorsotemporal part of the orbit and secretes via multiple excretory ducts into the conjunctival space beneath the lower eyelid (Harris *et al*. 2008; Jones, Pierce & Ward 2007; Kern 2007). The Harderian gland is located medioventrally to the eyeball, near the inter-orbital septum (Boydak & Aydin 2009; Burns 1992; Dimitrov & Genchev 2011; Kozlu & Altunay 2011; Mobini 2012).

In birds the lacrimal apparatus has been implicated as a part of the head-associated lymphatic tissue system (Rehorek *et al*. 2005, citing Burns 1992) in which the Harderian gland is the lympho-epithelial organ (Dimitrov 2011a; Ohshima & Hiramatsu 2002) and a site of immune response (Baba *et al*. 1990; Boydak & Aydin 2009; Kozlu & Altunay 2011; Payne 1994). The LG is responsible for production of tear fluid which, together with the Harderian gland and conjunctiva-associated lymphoid tissue, helps to maintain corneal health (Lavach 1990; Mohammadpour 2009).

The LG produces serous, mucous or seromucous fluid (Lavach 1990). The LG excretory products are part of the tear film that moistens and lubricates the anterior eye surface and also provides nutrients (Dartt 2009; Funaki, Hodges & Dartt 2010; Klećkowska-Nawrot & Dzięgiel 2008; Klećkowska-Nawrot *et al*. 2013; Payne 1994; Zagon *et al*. 2012). The tear film protects the anterior surface of the cornea and both superior and inferior conjunctival sacs (Jordan 1990; Payne 1994), and allows gas exchange between the air and the epithelium (Lavach 1990; Mohammadpour 2009). The tear film consists of three layers: a surface lipid layer, a middle aqueous layer, and an inner mucous layer (Walcott 1998) covering the exposed surface of the eye, namely the cornea and conjunctiva (Hodges & Dartt 2003). The LG produces the constituents of the aqueous layer (Hodges & Dartt 2003). The tears contain several soluble antimicrobial factors that protect the ocular surface (Davidson & Kuonen 2004). The LG-specific proteins found at highest concentrations in tears are lactoferrin, tear-specific pre-albumin and lysozyme (Kijlstra & Kuizenga 1994). A clear cornea with a smooth, well-lubricated facade is a prerequisite for clear vision. This is particularly important for animals that inhabit dry, hot and sandy areas (Mohammadpour 2009).

*Struthio camelus domesticus*, also known as African black ostrich, is usually referred to by breeders as ‘black necks’. The African black ostrich is the result of many years’ selection. This breed was created by crossing two subspecies: the North African ostrich, *Struthio camelus camelus*, and the southern African ostrich, *Struthio camelus australis* (Busse 1990; Horbańczuk 2003). African black ostrich is characterised by a milder temperament, better quality of feathers, as well as better growth than the others (Horbańczuk 1997).

Ocular diseases can have a significant impact on the condition and productivity of breeding animals. The majority of dry eye symptoms are the result of chronic inflammation of the LG, which decreases the ability of the eye to respond to environmental factors (Sorgolu, Yucel & Aktas 2003; Stern *et al*. 2004). The LG secretory function and tear film composition play an important role in eye physiology and pathology (Flanagan & Willcox 2009; Kawashima *et al*. 2012).

The aim of the present study was morphological and histochemical analysis of the LG during the embryonic and postnatal period in ostriches. Detailed knowledge of macroscopic and microscopic LG morphology can be a basis for diagnosis and treatment of ocular diseases in ostriches.

## Research methods and design

### Animals

The studies were performed on 50 African black ostriches aged between the 28th day of incubation and 7 months. The study included 12 pre-hatching birds (28, 33 and 40 days of incubation) and 38 post-hatching birds (24, 48, 72 hours after hatching; 2, 3, 5 weeks of age; 5, 6, 7 months of age). The animals were divided into four age groups on the basis of histological differences. The embryos were obtained from incubated eggs provided by an ostrich farm in Namyslow, Poland. Extracted embryos were classified into different developmental stages based on the days of incubation. The post-hatching birds from the ostrich farm in Namyslow were collected in 2010–2011. All the birds died as a result of natural causes. Mummified or underdeveloped embryos and nestlings were excluded from this study. The LG was dissected from embryos and birds immediately after death and fixed in 4% buffered formaldehyde for 48 hours.

### Macroscopic evaluation

Macroscopic evaluation of the LG was performed using a stereoscopic Zeiss Stemi 2000-C microscope (Carl Zeiss, Jena, Germany). Solutions of 0.5% – 4% acetic acid (CH_3_COOH), and 70% ethyl alcohol (C_2_H_5_OH) were used for the clear presentation of LG anatomical structure. LG morphology was described using topographic anatomical methods, namely holotypy and syntypy. Morphometric measurements (length and width) of glands were obtained using an electronic slide caliper with an accuracy of 0.1 mm. Data were statistically processed by statistical software (Microsoft Office Professional Plus 2010, Microsoft Corporation, Redmond, WA, USA).

### Histological analysis

The dissected LGs were immediately fixed in 4% buffered formaldehyde for 48 hours, rinsed in running water for 24 hours and then processed in a vacuum tissue processor (ETP) (RVG3, Intelsint, Italy), embedded in paraffin and cut on sliding microtome Slide 2003 (PfmA.g., Germany) into 3 µm – 4 µm sections. All samples were stained with H&E and Azan trichrometo demonstrate the general structure. The histological slides were examined with a Zeiss Axio Scope A1 light microscope (Carl Zeiss, Jena, Germany) for the histological description (Zawistowski 1965).

### Histochemical analysis

Histochemical analysis was conducted as follows: periodic acid-Schiff staining (PAS) to identify the presence of muco-substances (neutral or acidic) and glycoproteins; Alcian blue (AB) pH 2.5 to show sialylated glycoproteins; Hale's dialysed iron (HDI) and aldehyde fuchsin (AF) to demonstrate sulphated acid mucopolysaccharides (SAM) and carboxylated acid mucopolysaccharides (CAM) and elastic tissue was demonstrated by AF staining. Slides were examined with a Zeiss Axio Scope A1 light microscope (Carl Zeiss, Jena, Germany) with an Axio Vision system (Axio Scope A1, Carl Zeiss, Jena, Germany). PAS, AB pH 2.5, AF and HDI staining scoring systems were based on standard protocols that have been described previously (Spicer & Henson 1967). Anatomical terminology used was according to Paynter (1993).

## Results

### Gross anatomy

The LG of the ostriches became macroscopically visible by the 28th day of incubation, and appeared as a uniform, undivided and flattened gland. In all age groups this gland was bright red and oval, and positioned in the dorsolateral angle of the orbit, between the lateral and dorsal rectus muscles, close to the pyramidal third eyelid muscle and tendon of the pyramidal muscle. The LG secretes via multiple ducts that open into the conjunctival space beneath the lower eyelid. The nasolacrimal duct penetrated the lacrimal bone and rostrum maxillae premaxillae bone and opened into the nasal cavity.

### Group 1

#### Morphometry

**FIGURE 1:**
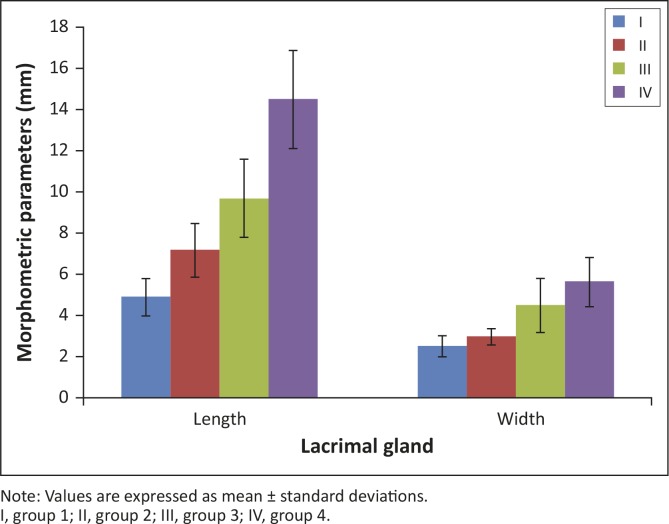
Morphometric parameters of the lacrimal gland in African black ostrich in the embryonic and postnatal period.Note: Values are expressed as mean ± standard deviations.I, group 1; II, group 2; III, group 3; IV, group 4.

The mean size of the LG in the first age group of pre-hatching birds (length × width with secondary duct [SD]) was 4.92 mm (± 0.9) × 2.65 mm (± 0.5) (Figure 1).

#### Histology and histometry

**FIGURE 2:**
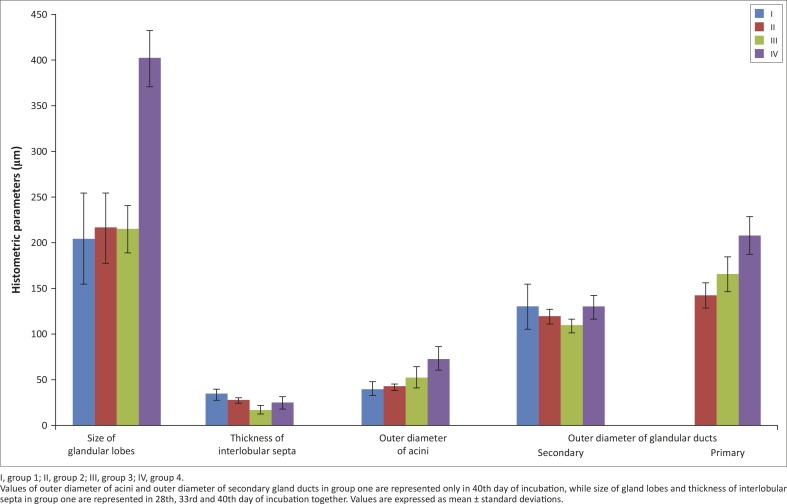
Histometric parameters of the lacrimal gland in African black ostrich in the embryonic and postnatal period.I, group 1; II, group 2; III, group 3; IV, group 4.Values of outer diameter of acini and outer diameter of secondary gland ducts in group one are represented only in 40th day of incubation, while size of gland lobes and thickness of interlobular septa in group one are represented in 28th, 33rd and 40th day of incubation together. Values are expressed as mean ± standard deviations.

**FIGURE 3:**
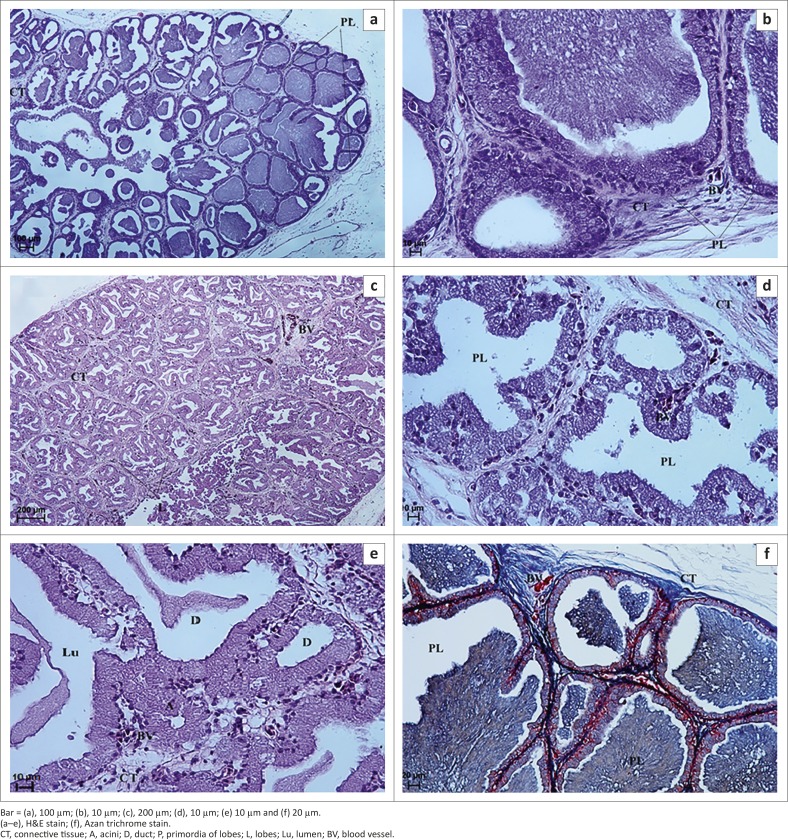
Light micrograph of the lacrimal gland in African black ostrich in group 1. (a) 28th day of incubation, (b) 28th day of incubation, (c) 40th day of incubation, (d) 33rd day of incubation, (e) 40th day of incubation and (f) 28th day of incubation.Bar = (a), 100 µm; (b), 10 µm; (c), 200 µm; (d), 10 µm; (e) 10 µm and (f) 20 µm.(a–e), H&E stain; (f), Azan trichrome stain.CT, connective tissue; A, acini; D, duct; P, primordia of lobes; L, lobes; Lu, lumen; BV, blood vessel.

**FIGURE 4:**
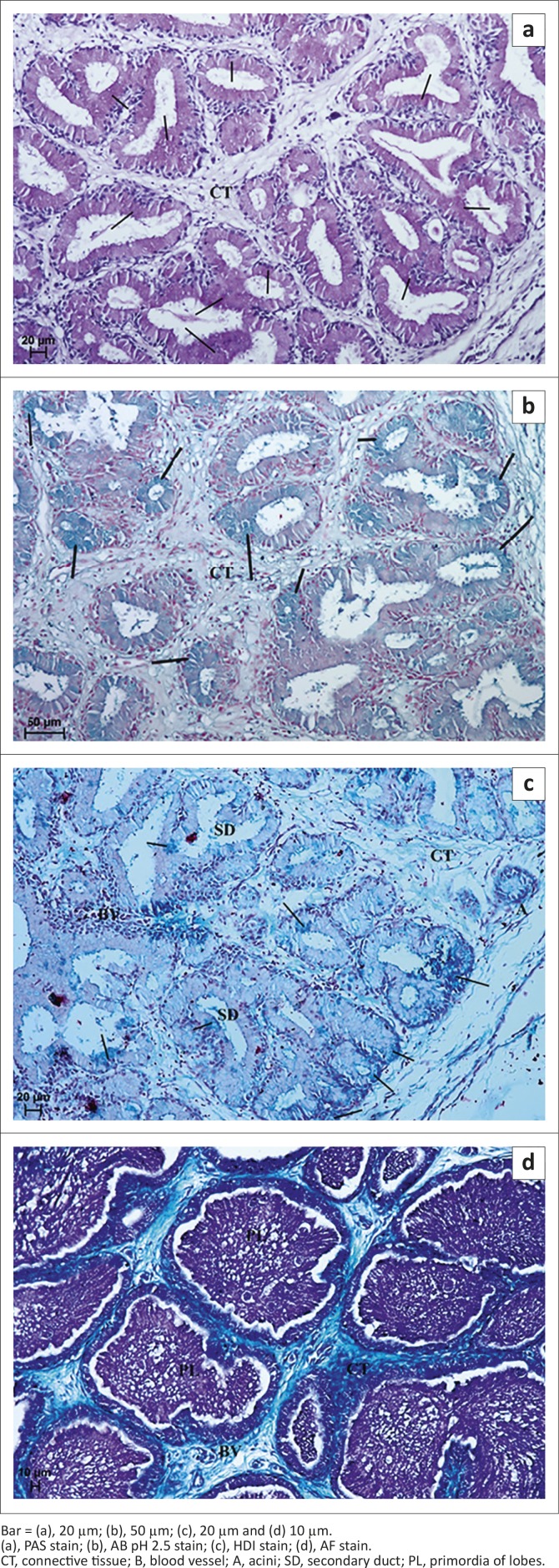
Light micrograph of the lacrimal gland in African black ostrich in group 1. (a) 33rd day of incubation, (b) 33rd day of incubation, (c) 40th day of incubation and (d) 28th day of incubation.Bar = (a), 20 µm; (b), 50 µm; (c), 20 µm and (d) 10 µm.(a), PAS stain; (b), AB pH 2.5 stain; (c), HDI stain; (d), AF stain.CT, connective tissue; B, blood vessel; A, acini; SD, secondary duct; PL, primordia of lobes.

The LG was covered by a thin connective tissue capsule with septa extending into the gland and dividing it into primordia or lobes (Figure 3a). The loose connective tissue was composed of adipocytes, a large amount of blood vessels, fibroblasts, rare lymphocytes, and collagenous and reticular fibres (Figure 3f). The numerous fine elastic fibres in the capsule and gland septa were demonstrated with AF staining (Figure 4d). The average thickness of connective tissue interlobular septa was 34.26 µm (± 6.5) (Figure 2). Amongst primordia of the lobes, lobules with cubic cells within the epithelial layer and a wide lumen were found on the 28th and 33rd days of incubation (Figures 3b and 3d). There was no division into acini and tubules (Figures 3c and 3f). The average size of lobes was 205.07 µm (± 49.6) (Figure 2). On the 40th day of incubation a clear division into lobes with interlobular connective tissue was observed, and the acini and ducts were present (Figure 3e). In the central part of the LG the lobes had 3–4 acini and tubules, whilst closer to the periphery of the gland the number of acini and tubules was higher (7–12). The mean outer diameter of the glandular acini on the 40th day of incubation was 40.7 µm (± 7.0) and the mean outer diameter of the secondary ducts was 130.61 µm (± 24.5) (Figure 2). The LG primary ducts were absent in the first group of birds.

#### Histochemical analysis

Examination of the LG with PAS staining showed numerous cells with a weakly positive reaction (−/+) that contained PAS-positive granules. PAS staining detected the presence of neutral and acid mucopolysaccharides and glycoproteins (Figure 4a and Table 1). AB pH 2.5 staining showed the presence of slightly positive granules (−/+) (light blue colour) in cells or single cells with blue granules that were considered positive (+) cells with AB pH 2.5 (Figure 4b and Table 1). This reaction indicated the presence of sialylated glycoproteins. HDI staining demonstrated a slightly positive reaction (−/+) in only a few cells, which were weakly blue in colour (Figure 4c and Table 1). HDI staining detects the presence of CAM. AF staining gave a positive reaction (+/++), with a purple colour that indicated the presence of SAM (Figure 4d and Table 1).

**TABLE 1:** Histochemical analysis of the lacrimal gland in African black ostrich in the embryonic and postnatal period.

Group	Method	Acini epithelium	Secondary duct epithelium	Primary duct epithelium
I	PAS	−/+	−/+	−/+
	AB pH 2.5	+	+	+
	**HDI:**			
	CAM	−/+	−/+	−/+
	SAM	–	–	–
	**AF:**			
	CAM	–	–	–
	SAM	+/++	+/++	+/++
II	PAS	+/++	+/++	+/++
	AB pH 2.5	+/++	+/++	+/++
	**HDI:**	++	++	++
	CAM	−/+	−/+	−/+
	SAM	–	–	–
	**AF:**			
	CAM	–	–	–
	SAM	−/+	−/+	−/+
III	PAS	+, +/++	+, +/++	+, +/++
	**AB pH 2.5:**			
	2 weeks old	−/+	−/+	−/+
	3 weeks old	++	++	++
	5 weeks old	++	++	++
	**HDI:**			
	CAM	++	++	++
	SAM	–	–	–
	**AF:**			
	CAM	−/+	−/+	−/+
	SAM	–	–	–
IV	**PAS:**			
	6 month old	−/+	−/+	−/+
	7 month old	+++	+++	+++
	AB pH 2.5	+++	+++	+++
	**HDI:**			
	CAM	++	++	++
	SAM	–	–	–
	**AF:**			
	CAM	–	–	–
	SAM	++/+++	++/+++	++/+++

PAS, period acid-Schiff; AB pH 2.5, Alcian blue; HDI, Hale's dialysed iron; AF, aldehyde fuchsin; SAM, sulfated acid mucopolysaccharides; CAM, carboxylated acid mucopolysaccharides.

### Group 2

#### Morphometry

The mean size of the LG in the second age group (length × width × with SD) was 7.22 mm (± 1.3) × 3.02 mm (± 0.4) (Figure 1).

#### Histology and histometry

**FIGURE 5:**
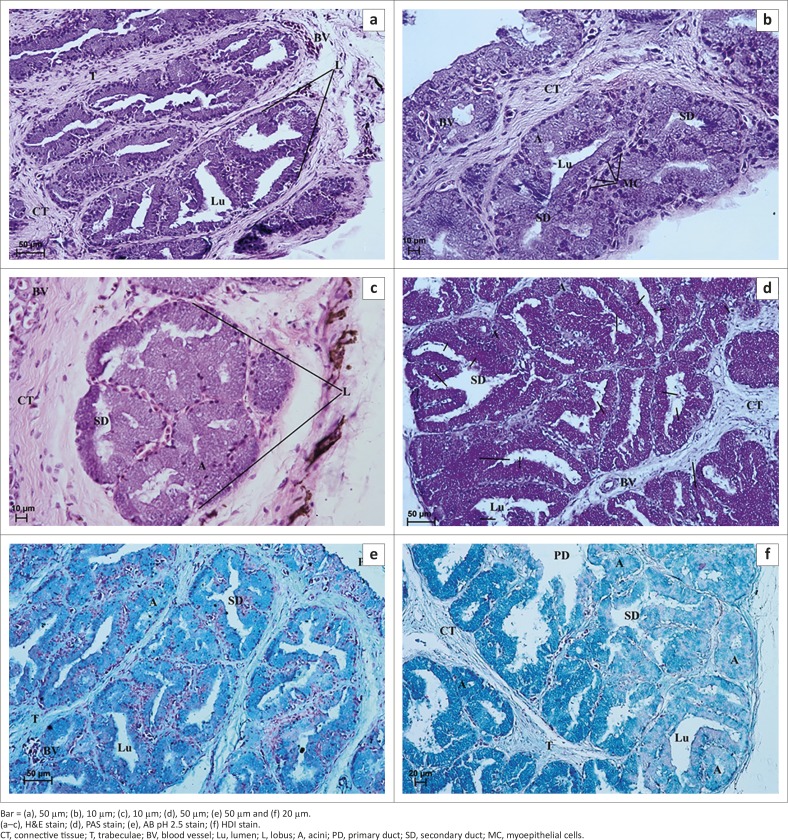
Light micrograph of the lacrimal gland in African black ostrich in group 2. (a) 24th hour after hatching, (b) 24th hour after hatching, (c) 72th hour after hatching, (d) 72th hour after hatching, (e) 24th hour after hatching, and (f) 48th hour after hatching.Bar = (a), 50 µm; (b), 10 µm; (c), 10 µm; (d), 50 µm; (e) 50 µm and (f) 20 µm.(a–c), H&E stain; (d), PAS stain; (e), AB pH 2.5 stain; (f) HDI stain.CT, connective tissue; T, trabeculae; BV, blood vessel; Lu, lumen; L, lobus; A, acini; PD, primary duct; SD, secondary duct; MC, myoepithelial cells.

In this group of birds the slightly marked lobes observed were comparable to those in the first age group. The average thickness of connective tissue interlobular septa was 27.57 µm (± 3.2) (Figure 2). The average size of lobes in this group was 216.91 µm (± 38.9) (Figure 2). Each lobe contained from 2 to 7 acini and ducts (Figure 5a). The primary and secondary ducts were well defined and branched (Figures 5b and 5f). The acini were composed of tall conical cells with a small lumen and were surrounded by myoepithelial cells (Figure 5c). The mean outer diameter of the glandular acini was 42.52 µm (± 3.4) (Figure 2). The ducts had one layer of cubic epithelium and a large and irregular lumen (Figures 5b and 5c). The mean outer diameter of the primary ducts in this group was 142.31 µm (± 13.7) and of the secondary ducts was 119.27 µm (± 8.6) (Figure 2).

#### Histochemical analysis

In this group of animals PAS staining of LG demonstrated the presence of secretory cells containing PAS-positive (+/++) granules irregularly located in glandular acini and ducts (Figure 5d and Table 1). Staining with AB pH 2.5 indicated the presence of positive granules (+/++) in glandular acini and duct epithelial cells (Figure 5e and Table 1). HDI staining showed a weakly positive reaction ( /+) in the peripheral part of lobes and a positive reaction (++) in the central part of lobes amongst the ducts, indicating the presence of CAM (Figure 5f and Table 1). In addition, the AF staining showed weakly positive granules (−/+) in acini and ducts, indicating the presence of SAM (Table 1).

### Groups 3 and 4

#### Morphometry

The mean size of LG in group 3 (length × width with SD) was 9.73 mm (± 1.9) × 4.54 mm (± 1.3) (Figure 1) and in group 4 was 14.52 mm (± 2.4) × 5.68 mm (± 1.2) (Figure 1).

#### Histology and histometry

**FIGURE 6:**
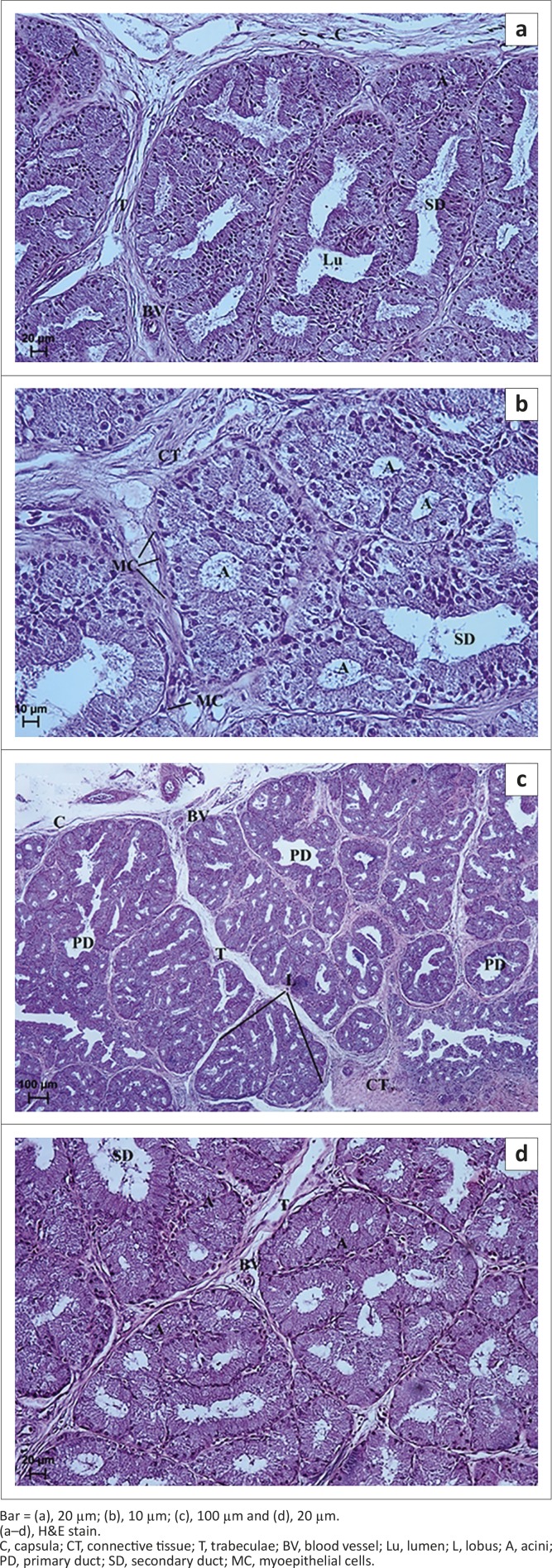
Light micrograph of the lacrimal gland in African black ostrich in group 3. (a) 2-weeks-old, (b) 2-weeks-old, (c) 3-weeks-old and (d) 3-weeks-old.Bar = (a), 20 µm; (b), 10 µm; (c), 100 µm and (d), 20 µm.(a–d), H&E stain.C, capsula; CT, connective tissue; T, trabeculae; BV, blood vessel; Lu, lumen; L, lobus; A, acini; PD, primary duct; SD, secondary duct; MC, myoepithelial cells.

Significant changes in the acini and secondary and primary tubules were observed in the group 3 and 4 birds. In these age groups less interlobular connective tissue and a clear division into lobes were observed in comparison to the first and second age groups (Figures 6a, 6c and 8a). The average thickness of interlobular septa in the third age group was 17.41 µm (± 4.9) and in the fourth age group 25.23 µm (± 6.7) (Figure 2). The average size of lobes in the third age group was 215.7 µm (± 25.8) and in the fourth age group 402.49 µm (± 30.8) (Figure 2). The acini were composed of tall conical cells that formed a small lumen and were surrounded by myoepithelial cells. The myoepithelial cells lay around the basal membrane of the acini (Figures 6b, 6d and 8c). The nuclei of the acini cells were ovoid and located in the basal compartment of the cytoplasm. These cells had granular, basophilic, vacuolated cytoplasm (Figure 6b, 8c and 8d). The mean outer diameter of the glandular acini in the third age group was 52.67 µm (± 11.4) and in the fourth age group 73.63 µm (± 12.7) (Figure 2). The secondary tubules were lined with cuboidal cells and had a large and irregular lumen. The nuclei of these cells had an oval shape and were located in the basal part of cells (Figures 6b, 6d and 8c). The mean outer diameter of the secondary ducts in the third age group was 109.28 µm (± 7.1) and in the fourth age group was 130.08 µm (± 13.1) (Figure 2). In the central part of each lobe from 2 to 4 primary ducts were present. The mean outer diameter of the primary ducts in the third age group was 165.75 µm (± 19.2) and in fourth age group 208.51 µm (± 20.6) (Figure 2).

#### Histochemical analysis

The differences in histochemical findings between the third and fourth age groups of birds are indicated in Table 1.

**FIGURE 7:**
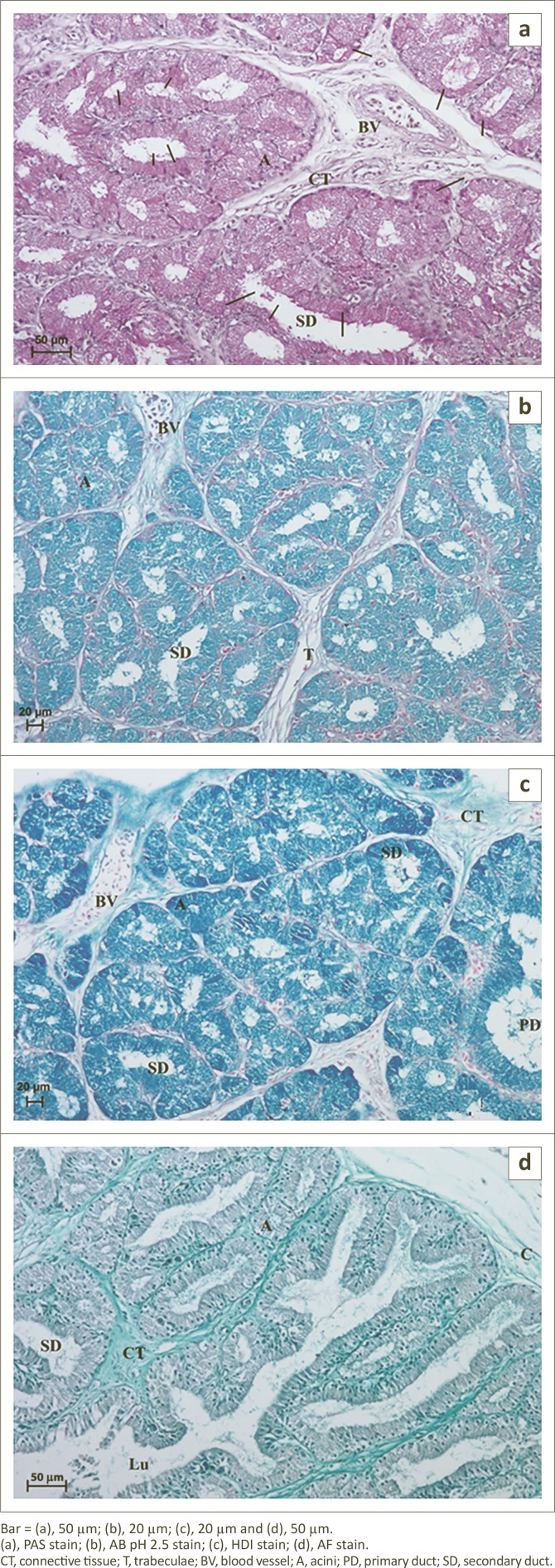
Light micrograph of the lacrimal gland in African black ostrich in group 3. (a) 3-weeks-old, (b) 3-weeks-old, (c) 5-weeks-old and (d) 2-weeks-old.Bar = (a), 50 µm; (b), 20 µm; (c), 20 µm and (d), 50 µm.(a), PAS stain; (b), AB pH 2.5 stain; (c), HDI stain; (d), AF stain.CT, connective tissue; T, trabeculae; BV, blood vessel; A, acini; PD, primary duct; SD, secondary duct.

**Group 3:** PAS staining demonstrated the presence of secretory cells containing PAS-positive granules evaluated as (+) and not many cells with a stronger reaction, evaluated as (+/++), irregularly located in glandular acini and primary and secondary ducts (Figure 7a and Table 1). Staining with AB pH 2.5 in the LG of 2-week-old ostriches demonstrated the presence of weakly positive granules (−/+) in the glandular units and ducts (Table 1), whilst in the LG of 3-and 5-week-old birds this staining showed the presence of positive granules (++) in all acini and ducts (Figure 7b). HDI staining showed the presence of positive granules evaluated as (++) both in acini and in secondary ducts, and indicated the presence of CAM (Figure 7c and Table 1). AF staining showed weakly positive granules (−/+) in acini and secondary ducts that also indicated the presence of CAM (Figure 7d and Table 1).

**FIGURE 8:**
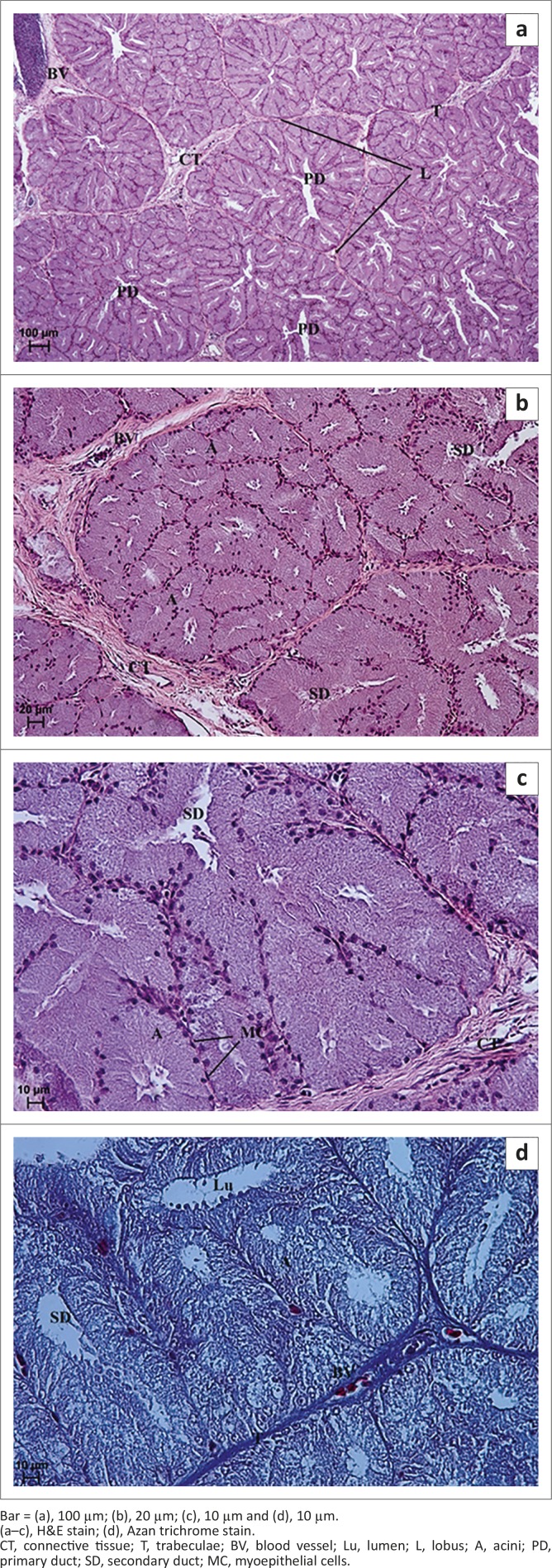
Light micrograph of the lacrimal gland in African black ostrich in group 4. (a) 6-months-old, (b) 6-months-old, (c) 5-months-old and (d) 7-months-old.Bar = (a), 100 µm; (b), 20 µm; (c), 10 µm and (d), 10 µm.(a–c), H&E stain; (d), Azan trichrome stain.CT, connective tissue; T, trabeculae; BV, blood vessel; Lu, lumen; L, lobus; A, acini; PD, primary duct; SD, secondary duct; MC, myoepithelial cells.

**FIGURE 9:**
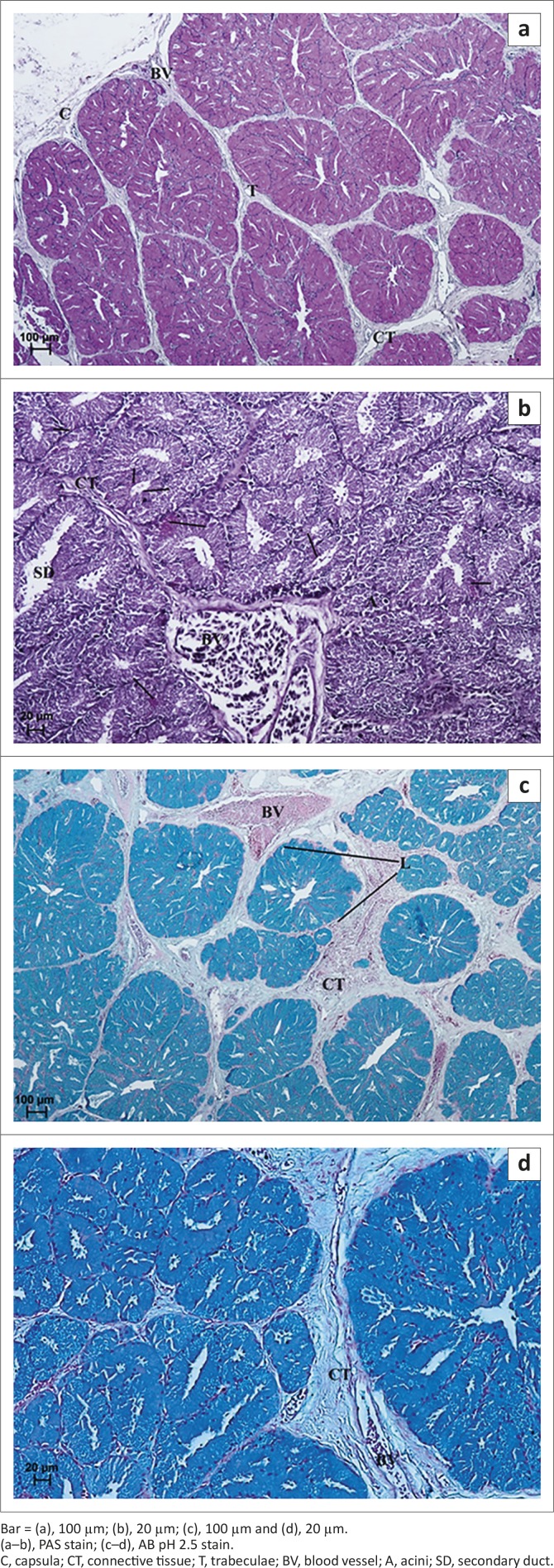
Light micrograph of the lacrimal gland in African black ostrich in group 4. (a) 7-months-old, (b) 6-months-old, (c) 6-months-old and (d) 5-months-old.Bar = (a), 100 µm; (b), 20 µm; (c), 100 µm and (d), 20 µm.(a–b), PAS stain; (c–d), AB pH 2.5 stain.C, capsula; CT, connective tissue; T, trabeculae; BV, blood vessel; A, acini; SD, secondary duct.

**FIGURE 10:**
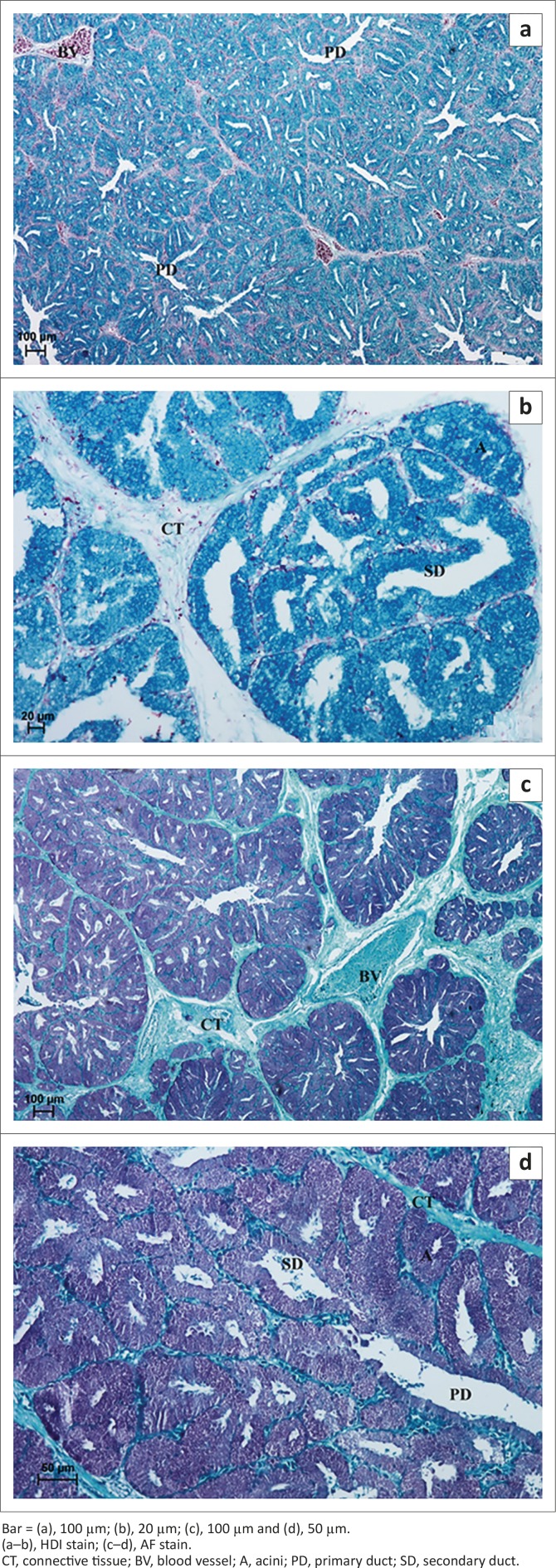
Light micrograph of the lacrimal gland in African black ostrich in group 4. (a) 7-months-old, (b) 5-months-old, (c) 6-months-old and (d) 5-months-old.Bar = (a), 100 µm; (b), 20 µm; (c), 100 µm and (d), 50 µm.(a–b), HDI stain; (c–d), AF stain.CT, connective tissue; BV, blood vessel; A, acini; PD, primary duct; SD, secondary duct.

**Group 4:** In 6-month-old birds a weakly PAS-positive (−/+) reaction was observed in a small number of glandular cells (Figure 9b and Table 1), whereas in 7-month-old birds a PAS-positive reaction, evaluated as (+++), was evident in all acini and ducts (Figure 9a). Staining with AB pH 2.5 indicated the presence of strongly positive granules (+++) located in glandular acini and primary and secondary ducts (Figures 9c and 9d, Table 1). HDI staining indicated a medium-positive reaction (++), similar to that in the third age group of ostriches, indicating the presence of CAM (Figures 10a and 10b, Table 1). In addition, the AF staining showed the presence of positive granules evaluated at (++/+++) in acini and ducts, indicating the presence of SAM (Figures 10c and 10d, Table 1).

## Ethical considerations

This study was approved by the II Local Ethical Committee in Wroclaw, Poland (No. 19/2010).

## Discussion

The LG is poorly described in birds compared to mammals (Burns 1976; Henker *et al*. 2013; Mohammadpour 2009). The LG gland in each age group of ostriches examined was located in the region of the posterior commissure of the eyelid, in the dorsolateral angle of the orbit between the lateral and dorsal rectus muscles, close to the pyramidal third eyelid muscle and tendon of the pyramidal muscle. Similar localisation of this gland has been demonstrated in other avian species, e.g. turkey, chickens and ducks (Burns 1979; Dimitrov 2011b). The LG in pigs is also located in the dorsolateral angle of the orbit, but between the dorsal oblique muscle and the lateral rectus muscle, and appears as a soft and pale structure (Henker *et al*. 2013), whereas the LG was bright red in the ostriches examined.

In the present study the ostrich LG appeared at around the 28th day of incubation as a uniform, undivided, oblate gland. Development of the LG is an example of an epithelial-mesenchymal interaction (Johnston *et al*. 1979). The tubular invagination, extension and branches give rise to the lobular structure of the mature gland (Kammandel *et al*. 1999). According to Chieffi Baccari, Di Matteo and Minucci (1995) the LG in the lizard *Podarcis s. sicula* appears on about the 22nd day of development. During development the acini fill up the preformed mesenchymal sac (Chieffi Baccari *et al*. 1995). The LG in pigs becomes macroscopically visible at the 42nd day of gestation (Klećkowska-Nawrot & Dzięgiel 2008).

Studies of the histological structure of the LG have been reported in various birds, but not in ostriches (Burns 1976; Dimitrov & Genchev 2011). In ostriches the LG was characterised by an increase in both length and width. The largest gland was observed in the fourth age group of ostriches, in which the largest increase in length was demonstrated compared to the other age groups. The entire surface of the LG was covered by a connective tissue capsule. The interstitium consisted of some collagen fibres separating the glandular lobe, as previously reported by others (Harris *et al*. 2008; Schechter *et al*. 2010).

In ostriches the LG consists of tubules lined with secretory epithelium and organised into lobes that drain into ducts; these anastomose into larger ducts that finally drain onto the ocular surface. The LG of the ostrich has been classified as a compound tubular-acinar gland, as in Japanese quails (Dimitrov & Genchev 2011). According to other authors the cells of the secretory epithelium in tubules and the acini are columnar with basally located nuclei. The duct cells are similarly organised, although more cuboidal in shape (Walcott 1998). Studies of the LG in turkey and chicken broilers demonstrated differences in the number of lobules between these birds (Dimitrov 2011b). In the present study the size of the glandular lobes was evaluated. Histometric measurements of glandular lobe size showed little difference amongst the first, second and third age groups; however, in group 4 a marked increase in lobe size was observed. Different results were obtained in measurement of interlobular septa thickness, which decreased from the first to the third age group, with an increase of thickness in the fourth age group. This may be related to the significant increase in lobe size in birds of the fourth age group. On the other hand, the outer diameter of acini showed an increase from the first to the fourth age group of ostriches. This increase in size was small in the first and second age groups and larger in the third and fourth age groups.

Varied results were obtained in measurement of the outer diameter of the primary and secondary glandular ducts. There were no primary glandular ducts in the first age group, whilst from the second to the fourth age groups a steady increase was demonstrated in the outer diameter of the primary ducts. Secondary glandular ducts were observed in each age group, but only from the 40th day of incubation in the first age group. The outer diameter of these ducts was high in the first age group. The outer diameter of the secondary ducts underwent a slight decrease in groups 2 and 3, increasing again in the fourth age group and reaching a size similar to the first age group. The number of secretory ducts in turkey and chicken broilers decreased from the periphery towards the central part of glandular lobules (Dimitrov 2011b).

There were no plasma cells in any of the groups of birds studied, and only a few lymphocytes were observed, confirming other reports that the Harderian gland is responsible for the production of immunoglobulin in birds (Khan *et al*. 2007; Kozlu & Altunay 2011; Nasrin *et al*. 2013). Different observations were made by Burns (1979). Surgical removal of the Harderian gland in the domestic fowl resulted in increased secretory activity of the LG and also in an increase in goblet cell numbers along the length of the LG duct. Plasma cells were more numerous in the LG in birds that were operated on (Burns 1979).

Histochemical analysis of ostriches’ LG showed differences in the proportion of secreted SAM to CAM in acini and duct epithelial cells. In each age group this proportion was evaluated on the basis of HDI and AF staining. SAM were indicated particularly by AF staining of the LG in the fourth age group of ostriches; however, SAM were not observed with either HDI or AF staining in the third age group. Furthermore, HDI staining did not demonstrate SAM in any age group of ostriches. Instead HDI staining showed low concentrations of CAM in the first and second age groups, and a higher concentration in the third and fourth age groups.

Very different results were obtained with PAS staining. In the LG of examined birds, PAS-positive cells were observed in each age group; however, only a small number of cells with a weakly PAS-positive reaction was demonstrated in the first age group. In the second age group a medium PAS-positive reaction was observed, but in the third age group the PAS-positive reaction was weaker. Similar results were obtained by Millar *et al*. (1996), who demonstrated a small number of PAS-positive acini cells in the superior and inferior LG of the rabbit. Results of research on morphological changes in rat LG indicate that ageing is associated with alteration in the ability of acinar cells to synthesise and secrete proteins (Draper *et al*. 2003). According to Sakai (1989) the lobules of the LG comprise a branched duct system and terminal acini with two types of secretory cells: acidic cells positive both for PAS and AB pH 2.5 and neutral cells positive for PAS and negative for AB pH 2.5. The AB pH 2.5 staining of the LG in examined ostriches indicated the strongest positive reaction in the fourth age group, both in acinar epithelium and in primary and secondary duct epithelium. In the same group a strong PAS-positive reaction was demonstrated in the 7-month-old birds. In the other age groups PAS and AB pH 2.5 staining showed a slight difference. Chieffi Baccari *et al*. (1992) also proved that the LG is composed of two cell types characterised by histochemical staining with AB pH 2.5 and PAS. Rare PAS-positive cells were found scattered in the acinar epithelium, as in the first age group of ostriches examined in this study.

## Conclusion

In the ostriches that were examined in this study, steady growth of the LG was observed with an increase in both length and width. Together with the growth of the gland, a marked increase in size of the lobes and glandular acini was observed, with apparent increase in dimensions of the gland structures. Within the lobes a linear increase in lobular size was observed. The development of primary ducts was present from the second to the fourth age groups. The study showed that apart from morphological changes, during the growth of LG the character of acid mucopolysaccharides varied. Histological study showed no plasma cells in any group of birds, and confirmed other reports that not the LG but the Harderian gland is responsible for the production of immunoglobulin in birds.
